# Potential educational and workforce strategies to meet the oral health challenges of an increasingly older population: a qualitative study

**DOI:** 10.1038/s41405-022-00098-5

**Published:** 2022-03-10

**Authors:** Georgina M. Prosser, Chris Louca, David R. Radford

**Affiliations:** University of Portsmouth Dental Academy, Portsmouth, PO1 2QG UK

**Keywords:** Gerodontics, Dental education

## Abstract

**Introduction:**

The demographic shift in the age profile of the UK is well established. Older people have more complex requirements to maintain their oral health.

**Objectives:**

This research explored Gerodontology with influential and recognised UK experts in Dental Public Health, Dental Education and Gerodontology. Three main topics were explored: the dental challenges associated with the ageing population, the role of dental care professionals (DCPs) and the training of undergraduate dental professionals.

**Methods:**

Quantitative data from the literature were used to develop a topic guide for semi-structured interviews with a purposeful sample of nine participants. Initial analysis of the qualitative data was undertaken using NVivo V12 software and themes were identified. Final themes and subthemes were confirmed after a series of remote round table discussions.

**Results:**

Four themes and 14 subthemes were identified. These included disagreements and agreements, the challenges of providing dental care to the older adult population, and the delivery of dental care by the dental team and finally education. All participants had significant concerns about the oral and dental challenges of the older population.

**Conclusions:**

The themes that emerged were complex and overlapping. Better utilisation of all members of the dental workforce was reported to be an essential approach, as was reorientation of existing care models with a focus on prevention. Regarding undergraduate education, the consensus was that the training was not adequate for both current and future needs.

## Introduction

The older adult population, defined as those 65 years and above, is projected to reach 1.6 billion globally by 2050 [1]. Steady ageing has been reported in developed countries over the last century, including the UK, owing to a decrease in birth rates combined with an increase in life expectancy [2]. In developed countries, the proportion of older people is increasing more than any other age group [3]. These demographic changes will have an increasingly significant impact on a range of economic, health and social policies [4]. Older people have a higher prevalence of chronic diseases and oral diseases as they share common risk factors and social determinants with chronic non-communicable diseases [1, 3]. Greater tooth retention is also increasingly common, which presents a challenge to both the individual and professional, and further it is reported that dentate older adults can require extensive and complicated dental treatment to maintain their dentitions [5].

Suboptimal oral health and complex treatment needs are not the only challenges. Oral diseases remain prevalent and under-diagnosed because older adults often do not receive routine dental care due to barriers and misconceptions. These barriers are divided into three categories: patient related, professional related and policy related [6]. There is agreement in the literature that the potential mechanisms to improve the oral health of the older adult population and approaches to overcome these barriers include: aligning health systems and ensuring access to quality services providing older person-centred care, creating age friendly environments, improving research on the healthcare needs of older people and building a sustainable and appropriately trained interdisciplinary workforce [7]. Despite these recommendations, there is a lack of current qualitative research exploring the thoughts of influential opinion leaders with regards to education in Gerodontology and how potential improvements could be made both in the UK and as a model to other countries to meet the significant challenges that the increasingly older population present. The aims of this research were therefore to qualitatively investigate the over-riding issues, educational and workforce strategies required to address the oral health challenges of the increasingly older adult population.

## Methods

Quantitative data from previous research on this topic was used to develop a topic guide for semi-structured interviews (Prosser G, Radford D, Louca C. Teaching of Gerodontology to Dental and Dental Hygiene Therapy students in the UK. Manuscript submitted for publication *Br Dent J*. 2022) [8]. The interviews with a purposeful sample of prominent influential and recognised UK experts, many with a global perspective of Gerodontology, Dental Education and Dental Public Health, explored three main topics: the dental challenges associated with the ageing population, the role of Dental Care Professionals (DCPs) and the current training of undergraduate dental professionals in the dental care of older people. The participants were emailed and invited to participate if they met the inclusion criteria. Eleven participants were approached and nine accepted the invitation, six were from a dentist and three from a dental hygiene therapy (DHT) background (Table 1).

**Table 1:** Participant background and expertise.

Participant number	Participant background	Expertise
1	DHT	Involved with training of undergraduate DHT students
2	Dentist	Retired General Dental Practitioner and expert in Gerodontology
3	Dentist	Professor and Consultant in Restorative Dentistry
4	Dentist	Consultant in Restorative Dentistry and involved with training of postgraduate dentists and DCPs
5	Dentist	Professor and Consultant in Dental Public Health
6	DHT	Involved with training of undergraduate DHT students
7	Dentist	Involved with training of undergraduate DHT students
8	Dentist	Involved with training of postgraduate dentists and DCPs
9	DHT	Involved with training of undergraduate DHT students

Ethical approval was gained from the University of Portsmouth (SHFEC 2021-002) and the interviews were conducted over Zoom due to Covid-19 restrictions. The interviews were undertaken at a time of the participants’ choosing and took place over a two-month period from March to April 2021. The participants consented and undertook a semi-structured interview with one of the researchers (GP) and the interviews did not have a time limit. The mean length of the interviews was 30 min, and the range was 22 min to 40 min. The interviews followed a defined set of topics that had been discussed by all three authors (GP, CL and DRR) and the participants did not see the topic guide prior to the interview. Two of the researchers (CL and DRR) have previously worked with the participants in the study. GP is a Dental Core Trainee and DRR is a former reader and Restorative Consultant and a specialist in removable prosthodontics.

The interviews were recorded and transcribed verbatim (GP). The subsequent transcripts were then anonymised and analysed by one researcher (GP) and transcripts sent to the participants to check on correct interpretation prior to the analysis. After familiarisation, thematic analysis of the qualitative data was undertaken using NVivo V12 software. Initial codes were generated with a subsequent search for themes. The themes identified were then reviewed and defined by name. A second independent analysis of the transcripts was undertaken by an experienced qualitative researcher (DRR). The final themes and subthemes were finalised between the lead researcher GP and DRR over five remote round table discussions. During these discussions both researchers were reflective of their personal bias due to their background and previous experience.

## Results

**Fig. 1:**
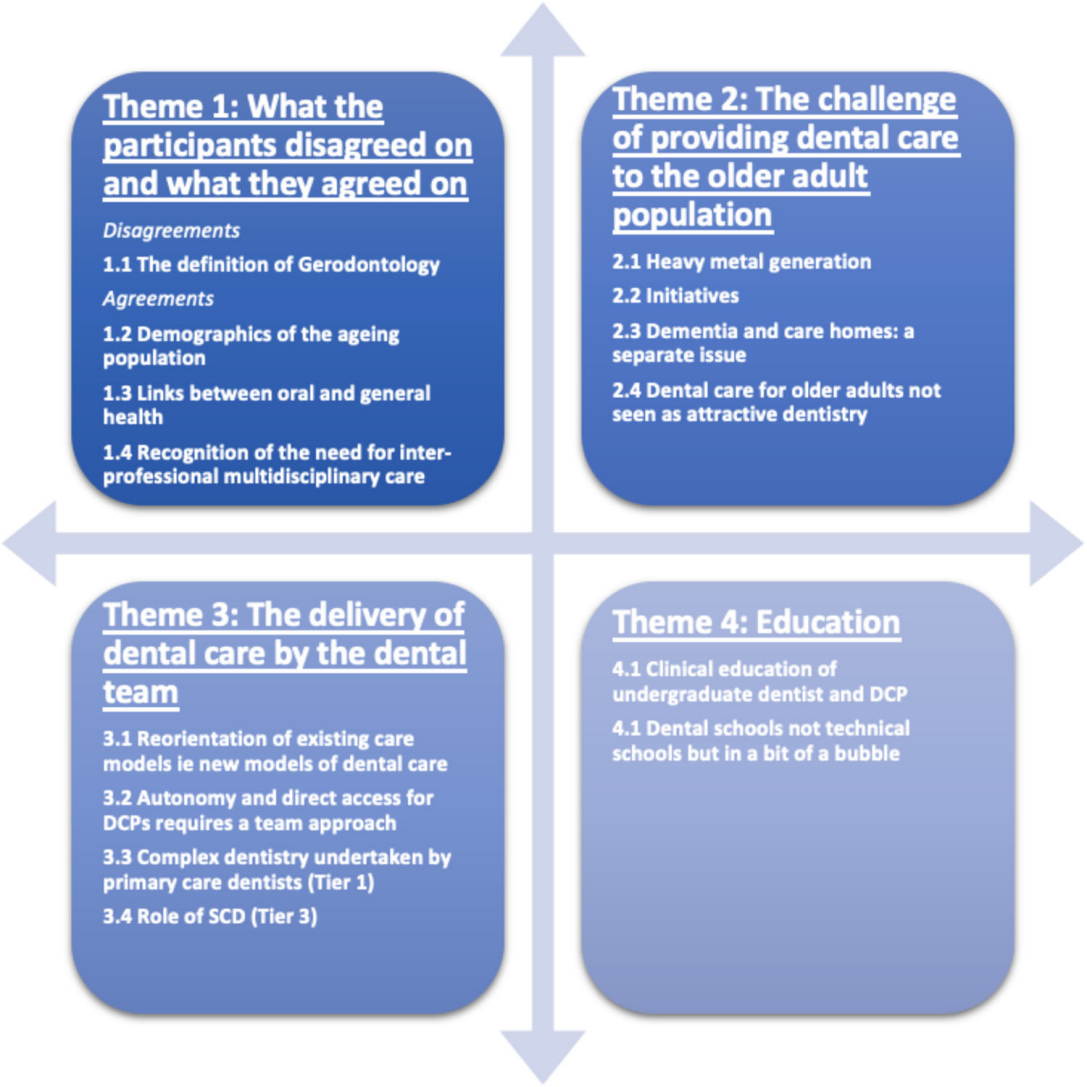
Themes and subthemes.

The four themes were identified (Fig. 1).

### Theme one: What the participants disagreed on and what they agreed on

Theme one explored what the participants disagreed on and what they agreed on. There was disagreement about the definition of Gerodontology with participants claiming the definition to be vague and poorly described in the literature.

### Disagreements

#### The definition of gerodontology

*“It [Gerodontology] has never been properly defined, and it is defined variably by different people, by different organisations, and in the literature, you can find several definitions of it” (Participant 3, line 244–246 on the transcript) therefore this will be annotated as (P3, 244–246)*.

All participants agreed on the challenges of the ever-increasing number of older adults and commented on the close link between oral and general health and the resultant need for inter-professional multidisciplinary care.

### Agreements

#### Demographics of the ageing population

*“A decline in the number of people with complete dentures and so that’s an increase in the number of people with teeth… and the ONS [Office for National Statistics] data will presumably say that the numbers of older people are also increasing… so… older people have a need which is not being fulfilled by the curriculum that undergraduates currently undertake” (P2, 356-361)*.

*“The demographic detail is very clear, a growing number of older people… they are retaining their teeth longer into old age and that brings with it huge new challenges for dentistry. Just the sheer numbers, let alone if you factor it up, as I always say to heart surgeons you know [sic] there’s going to be 5,000,000 more old people that’s only 5,000,000 more hearts but if old people may have 20 teeth, that’s 100,000,000 more teeth to look after” (P3, 10-16)*.

#### Links between oral and general health

*“It’s still… on the periphery of the General Healthcare radar screen and I think dentistry collectively, the whole dental team, we’ve got to get the rest of healthcare to take us more seriously and to better understand the now irrefutable link there is between oral and dental health and general health and wellbeing. That’s particularly true in older people, you know, if they’ve got uncontrolled unmanaged periodontal disease, and the links with cardiovascular, cerebral degeneration” (P3, 334-340)*.

*“The… sooner we put the mouth back in the body the better, so that medics understand how important dentistry is in a whole host of stuff” (P8, 290-292)*.

#### Recognition of the need for inter-professional multidisciplinary care

*“In terms of them joining up with the allied medical professional colleagues, I would support that widely and I think there’s examples where that’s happening for instance we have… our dental hygiene and therapy foundation training already going in and visiting NHS [National Health Service] clinics etc, where they will be coming into exposure with medical allied professionals” (P4, 331–335)*.

*“Actually, then when it gets to the point that people have multiple comorbidities then perhaps the complexity is greater, perhaps its dementia and other challenges… that’s where it’s quite often useful to have somebody with the necessary skills and expertise within the team, and to liaise… with other members of the health and social care system” (P5, 102-103, 105-108)*.

### Theme two: The challenge of providing dental care to the older adult population

The second theme was the challenge of providing dental care to the older adult population. Many participants referred to this patient cohort as the ‘heavy metal generation’ as they have a heavily restored dentition with amalgam restorations. Further older people are retaining more of their natural teeth so participants reported that there will be an increased demand for more complex dental procedures such as the management of tooth surface loss and provision of indirect restorations. Some reported that the expectations of older people are also likely to have increased as many will have paid for implants and cosmetic dental procedures, so they will not just want a functional dentition but also for it to be aesthetically pleasing. There was, however, agreement that for many older adults the goal is to maintain a functional pain free dentition through prevention and maintenance.

#### Heavy metal generation

- Expectation of teeth for life but not a perfect dentition
- Maintenance of everyday brushing, fluoride, early root caries

*“I think their expectations are going to grow too… give them another 10 or 20 years, you’re going to start getting older people that have spent a lot of money on adult orthodontics, bleaching, aesthetic dentistry, that would like to continue to remain looking young and youthful etc, and will continue to expect that in their advancing years” (P3, 67-72)*.

*“We’re not looking for perfect oral health but we want people to live in comfort” (P5, 207-208)*.

*“It is very much seeing the older population with you know [sic] sort of this heavy metal generation and I think it’s going to be a real challenge of how we manage patients who you know are taking multiple different prescriptions for various things that cause the dry mouth and therefore the root caries and also you know quite a heavily restored mouth from you know earlier in life, so I think it’s going to be a really big challenge for us and whether we are equipped to do it I don’t know” (P6, 23-29)*.

Many UK initiatives were discussed including the ‘Advancing Dental Care review’, which aimed to develop an education and training infrastructure that could respond to the changing needs of patients and services. An Oral Health Practitioner (OHP) is an individual with specific training that can perform a range of oral health preventive procedures, involving working in a patient’s mouth, and the OHP apprenticeship was one of many schemes piloted in the UK. Another initiative was ‘Mouth Care Matters’ which is a UK programme that aimed to improve the oral health of hospitalised adult patients.

#### Initiatives

##### Advancing dental care

*“Advancing dental care really looked at the whole picture, yes we’ve come up with the Oral Health Practitioners as a new model, yes we’re looking at how we can use therapists more effectively and also looking at the whole of core training and specialty training” (P8, 101-105)*.

##### Mouth care matters

*“We have foundation dentists doing mouth care matters, we could equally well have therapists doing mouth care matters with a particular emphasis on doing the routine cons that they could do for the older patients” (P8, 216-219)*.

##### Oral health practitioner (OHP)

*“I think it [the Oral Health Practitioner Apprenticeship] is one of the things that could be a gateway if you like, to multi professional working so you’re going to have, instead of the dentist wondering how they’re going to cope with domiciliary visits, you do have regular contact with some of these patients through your Oral Health Practitioner” (P8, 49-53)*.

##### Coordination of initiatives

*“There has to be I think, a clear directive or initiative or demonstration of how this can all work as a business model and actually pilot it and run it through to show the risks and benefits with it” (P1, 152-155)*.

Participants often separated older patients into groups based on their dependency level and the dependant group, often in residential care homes, and those with cognitive impairments such as dementia, were seen as very much separate from the largely fit and healthy older cohort.

#### Dementia and care homes: a separate issue

*“On top of that you then have the whole issue around dementia and other comorbidities, complex health issues, and then on top of that you’ve got if you’re housebound or care home bound, how do you necessarily deliver the treatment” (P8, 20-24)*.

*“You’ve got to have the communication skills and when you’ve got people with dementia or people who are a bit older don’t hear well, the communication is key, so you’ve obviously got to be able to explain to the patient and maybe with the carer” (P8, 242-246)*.

Many participants shared the concern that dental care for older adults is not seen as attractive dentistry for younger dental professionals who are more interested in cosmetic procedures.

#### Dental care for older adults not seen as attractive dentistry

##### Higher interest in facial aesthetics, whitening, dental implants

*“People would rather be doing Masters in Restorative dentistry… or cosmetic dentistry or children’s dentistry, Orthodontics… maybe that’s because it’s where the money is, I don’t know” (P2, 263-266)*.

*“How do we on the one hand train our profession and place them to deliver care… where in the background they might want to do a lot more around the younger patient group… whitening of teeth, implants, Invisalign [alignment of teeth]. I don’t quite know how we are going to square that with our current models” (P4, 76-81)*.

### Theme three: The delivery of dental care by the dental team

Theme three concerned the delivery of dental care by the dental team and was the largest and most complex theme. Participants agreed that a reorientation of existing care models was necessary with a greater focus on prevention and better incentivisation, especially for DCPs.

#### Reorientation of existing care models i.e., new models of dental care

*“My hope is that we just don’t think we can use the DCP model, changing diagnosis ability, etc, to try and fill the gap that actually the NHS and the dental profession should be addressing, and that’s my fear, that we are using one solution to what is a very complicated problem” (P4, 355-359)*.

*“It’s only one of a series of changes, we don’t want… to just give them [DCPs] a performer number and almost make them like mini dentists is not changing the system” (P5, 211-213)*.

*“I think we’ve got to think creatively about new models of care, that does require us to think about funding systems and it requires courage from our leadership and support from politicians ultimately to achieve change and people have got to value dentistry” (P5, 300-304)*.

*“I think we do have one of the best models in the world and we just don’t use it properly… it’s really about utilisation and education” (P9, 268-270)*.

##### Issue of private vs NHS dentistry

*“I think one of the big arm wrestles we’ve got in dentistry is that we’ve got the private market and we’ve got the NHS market and if we accept the NHS market is a finite resource, we need to focus our attention on the most difficult and most challenging cases and the trouble with that is that’s politically a nightmare, politicians don’t like to see rationing of healthcare” (P4, 67–71)*.

*“The NHS system, you know band 1, band 2, band 3 [this is the methods of remuneration in the current NHS contract for dentists and is determined by the level of complexity of dental care], doesn’t really work because people need so much more time” (P7, 18-19)*.

##### Need for better incentivisation for all members of dental team, especially DCPs

*“I think we’ve got people already on the registers, it’s a question of getting them to work but there needs to be enticements for them to work in the way that we all think they should be working” (P4, 121-123)*.

*“I think… what the performer number will give them… it’ll give them you know sort of terms and conditions of employment so it will give them access to NHS pensions… proper maternity leave” (P4, 134-137)*.

*“The problems that we have in dentistry are focused to pockets of deprivation, so the problem we’ve got is how do we adequately resource those pockets of deprivation that need most resource” (P4, 346-348)*.

*“So, it may well be that the way to deal with the ageing population is to incentivise GDPs to pay more attention to it by enhancing their contract with this transformational commissioning model, so you would reward a practice for employing an oral health practitioner” (P8, 64-67)*.

Participants were in favour of direct access, however, only in a supportive functional team setting with overall responsibility taken by an appropriately qualified dental professional. Direct access in the UK is where DCPs, namely dental hygienists, dental hygiene therapists and Clinical Dental Technicians (CDTs) for complete dentures only, can carry out their full scope of practice without prescription and without the patient seeing a dentist first [9]. CDTs are dental technicians that have undertaken further training in order to “prescribe and provide complete dentures direct to patients and provide and fit other dental devices on prescription from a dentist” [10].

#### Autonomy and direct access for DCPs requires a team approach

##### Good for primary care – patients not concerned with who they see, often more happy / better relationship with DCP

*“From a personal perspective, my patients always enjoyed seeing me more and they would try to avoid my boss as much as possible. They knew if they saw me nothing was wrong and they were holding on steady state, that is one way of looking at it” (P1, 164–167)*.

*“I think many older people… might be happier being referred to a therapist or hygienist who may have a bit more time for them, who will chat a bit more maybe” (P3, 140, 143–145)*.

##### Less good for nursing homes

i. OHPs for prevention
ii. DCPs for prevention and maintenance

*“[When asked if dental therapists are adequately trained to visit care homes provide oral healthcare] Routine oral healthcare and screening… I would say yes, you know but… as part of a dental team when there is a dentist you know backing up or maybe visiting the care home every two years or something” (P3, 229–232)*.

##### DCPs not cheap dentists and play a major role in prevention

*“We need to think about what people are delivering and what is best for the patients and is it contemporary preventatively orientated minimally invasive dental care, and if you move hygienists [and] therapists almost into being cheap dentists, which you know some people think is the way forward, who is going to do the prevention?” (P5, 214–218)*.

*“I think therapists have got all of the clinical skills in managing, like we said, root caries or general caries but also all of the preventative aspects… I think we [DCPs] have a very strong role to play in older care” (P6, 98–100, 103)*.

##### Clinical dental technician (CDT)

*“The trouble with the dental therapy and the [clinical] dental technician… somebody’s got to take ownership for treatment planning” (P4, 160-161)*.

*“Clinical dental technicians can play a role, but they tend not to be located in the areas where perhaps the population might need them… in terms of making partial dentures they’ve got to work with other members of the dental team and… so far research has shown… they are not well enough understood in their roles” (P5, 141–143, 144–146, 148–149)*.

Individuals claimed that complex dental procedures should be undertaken by primary care dentists (Tier 1), however, specialties such as special care dentistry (Tier 3) may be required in certain situations. These three tiers or levels were outlined in the ‘Introduction Guide for Commissioning Dental Specialities’ document published by the National Health Service (NHS) in 2015. Level one complexity was defined as the “skillsets and competencies that are covered by the teaching and training in the dental undergraduate and Dental Foundation programme”, level two as “procedural and/or patient complexity requiring a clinician with enhanced skills and experience who may or may not be on a specialist register” and finally level three as “care that requires specialist practitioner or consultant led care due to complex clinical or patient factors” [11].

#### Complex dentistry undertaken by primary care dentists (Tier 1)

*“In old age, you hopefully aren’t going to get much caries in the healthy person but when teeth crack and fracture and suffer wear and tear, you’re looking at really more advanced forms of operative conservative dentistry to manage them and I’m not sure that it’s fair to expect therapists to deal with some of those issues, they may not have been trained to deal with that extent of damage and wear and tear to teeth” (P3, 109–115)*.

#### Role of special care dentistry (Tier 3)

*“I think it should be, but specialists should only be treating the very complicated so… in England we have commissions standards of one, two and three so Special Care Dentists should be dealing with tier three patients, complexity three, and they should be providing… treatment plans etc for others” (P4, 86–90)*.

##### One off episode of treatment then back to primary care

*“All specialties are meant to be seeing the more complex end of the spectrum, the sort of thin end of the wedge, there are patients who might occasionally be complex so it might be that one episode of care is complex but actually the rest can go back into a primary care setting” (P5, 67–71)*.

##### Special care already overloaded with other patient groups

*“Specialists should only be treating the very complicated… and they should be… overseeing treatment plans etc for others but in terms of Special Care Dentistry… especially on its own managing this problem, it would not touch the sides” (P4, 86–87, 89–91)*.

*“We know the community dental services are too stretched to do it” (P6, 193–194)*.

### Theme four: Education

Theme four centred on education. Many participants reported that the already significantly overcrowded curriculum for both dentists and DCPs is the main barrier preventing the adequate level of training and education in the dental care of older adults, not a lack of awareness of the educators. All participants did agree that more intra-professional (within the dental profession) and inter-professional (from two or more professions) training was essential to prepare the workforce for the ideal multidisciplinary holistic approach, however, often logistics made this difficult to achieve. Many reported that side by side training from the start between dentists and DCPs will help to break down barriers and preconceived attitudes and therefore will result in a workforce that is more aware of the specific skills and scope of practice of each type of dental professional.

#### Clinical education of undergraduate dentist and DCP

*“For hygienists and therapists, I think it’s important, dental nurses and anybody who is willing to get involved in somebody’s mouth, they understand the implications of ageing and the complications of ageing, that there is still a person inside there… It’s important that they are educated about ageing, the ageing process and the pathology which goes with ageing, which in my mind are two separate things” (P2, 59–62, 64–66)*.

*“Dental students on the whole are not well trained in caring for older people… there’s no problem with people, older people who enjoy good general health and mobility and so on because it’s just like treating any other adult, [they] maybe need a bit more time… The training for the older person who has you know lots of medical and associated complications, particularly mobility or social care issues etc, on the whole it is variable between dental schools, some dental schools have been good at it… other dental schools… in the best of my knowledge, are not strong enough” (P3, 256–259, 261–264, 267–268)*.

*“Given there’s going to be more older people and older people have been kept alive because of the modern-day medicine etc… you probably need some more [Gerodontology training] but… what do you take out” (P3, 270–273)*.

##### Intra-professional training

*“I would say we need even more integration in training schools… [like what] happens at Portsmouth [University of Portsmouth Dental Academy] etc, that you know, DCPs are working together with trainee dentists and are working as a team” (P3, 359–361)*.

*“I really favour it, joint learning and working is the key… then you breakdown any preconceived attitude’s… between dentists and dental therapists and dental nurses and hygienists and I’m afraid there is still a lot of barriers there and the barriers are two ways, it’s not just dentists towards DCPs, it’s also DCPs to dentists” (P4, 280–284)*.

*“It starts at the beginning doesn’t it, so basically side by side training at the beginning. So, you’re starting with undergraduate or pre-registration training aren’t you and then you’re moving right the way through the system and the NHS workplace” (P4, 297–300)*.

##### Lectures and inter-professional education

*“We are looking to do a lot of inter-professional training… we are now looking at it in the far bigger medical picture and actually a human being rather than a simulation head” (P1, 293, 295–296)*.

*“I agree philosophically, I think practically it’s not always as easy as it sounds, some people, places have got it to work well, others not” (P5, 281–282)*.

Alongside comments on the need for more inter-professional and intra-professional education embedded in programmes of study for all members of the dental team, participants also expressed the view that there should be additional training opportunities to provide dental care outside the dental hospital as this is not representative of primary care settings.

#### Dental schools are not technical schools but in a bit of a bubble

##### Need outreach and primary care

*“Delivering that [dental care] here in the hospital or hopefully out in some primary care placements where they will get it in real life rather than in the hospital bubble” (P1, 228–230)*.

*“Don’t forget everybody nowadays is prepared and are not just trained, they are educated and trained because… you’re not going to a Technical College to get training at undergraduate level, its education and training… so they will be educated and trained to be safe beginners” (P5, 175–178, 179–180)*.

##### Need to incorporate care homes

*“My impression would be that neither dental students nor dental hygienists nor dental therapists get any training whatsoever in domiciliary dental care and again… that’s a little bit sort of specialist for the undergraduate curriculum, whether they ought to or not is a different question but at the moment I would say they are not” (P2, 188–192)*.

## Discussion

The demographic shift in the age profile of the UK is clear [12]. The dental professionals interviewed in this study, were concerned about this demographic change and the considerable challenges that the increasingly older population will present. The participants appeared to categorise the challenges into two distinct areas: dental related and patient related.

Regarding dental care, participants referred to the ‘heavy metal generation’ and the resultant increased complexity of dental care, however, there was agreement that for many older adults the goal is to maintain a functional pain free dentition through prevention and maintenance. A few participants reported that this demand for advanced restorative dental care should be similar to the care required by working age adults, if the individual is still independent and able to attend a primary care setting. Where this is the case, they reported that primary care dentists can provide this restorative care and in cases where it is too advanced, a referral to a mono specialist dentist would be appropriate. It is true that with older age the incidence of common, mild and major systemic diseases increases and this can result in polypharmacy for many older adults. Whilst it is still appropriate to treat these individuals in primary care, all dental professionals will need to have an awareness of these conditions and medications and manage these patients appropriately.

The major concern for participants focused, however, on the frailer older patient group, often with multiple comorbidities and functional dependency, and those living in residential care homes. Although those in residential care only account for a small percentage of the over 65 population, the numbers are still significant. In 2018, 400,000 older people were living in care homes, 70% of whom also had dementia or severe memory problems [13]. Participant eight commented that in these care homes there is uncertainty regarding the delivery of dental care. Participants generally shared the notion that this category of patients may initially need input from a Special Care Dentist, however, a few were concerned that this speciality is already over committed with other patient groups. One participant (P8) offered the solution that after this initial episode of treatment, the remaining care, which should consist of prevention and maintenance, could be passed onto less highly trained individuals who are incentivised appropriately to care for this patient group.

It is evident that there is a great variation in both the health and dental status of older adults, and this is largely irrespective of chronological age. This makes it complex to consider older adults as a “one patient demographic” as their needs are wide-ranging and patient specific. This is one of the criticisms the participants had with ‘Gerodontology’ as a distinct speciality as it significantly overlaps with other specialities, as well as it being poorly defined in the literature. In this study, it became clear that the term ‘Gerodontology’ meant different things to different people. Some participants agreed that it is anyone over the age of 65, however, others saw it as a subsection of Special Care Dentistry dealing with older patients with additional needs. For this reason, further consensus across experts is required to clarify the definition of Gerodontology, which would be beneficial and result in the profession better able to lobby for appropriate resources to be targeted at the older adult patient group that is being failed by the current system.

It is common knowledge that oral diseases share common risk factors and social determinants with non-communicable diseases such as cardiovascular disease, hypertension, and diabetes, and so tend to be more prevalent in patients with these comorbidities [1]. Poor oral health can contribute to reduced chewing ability, constrained food choice, weight loss, impaired communication and low self-esteem, which have been documented to reduce the quality of life [14]. Oral health tends to be particularly suboptimal in the medically compromised and functionally dependant older adults, and it has been reported that neglected oral hygiene and tooth loss may increase morbidity [15]. All participants shared this appreciation and reported that a multidisciplinary approach is essential to achieve general health and wellbeing. Many, however, were frustrated that currently the dental speciality is rarely included within this inter-professional team. One participant (P3) even exclaimed that healthcare needs to take dentistry more seriously and understand the importance of the links between general and oral health.

A multidisciplinary approach was only one possible solution to the challenges that the older population present, and others discussed innovative initiatives such as Advancing Dental Care which aimed to “develop an education and training infrastructure that could respond to the changing needs of patients and services” [16]. The concept of an ‘Oral Health Practitioner’ is another demonstration of how the dental team can be employed more efficiently.

As discussed previously, many participants shared the view that independent older adults can be treated in primary care just like any working age adult as the dental care required is no different. The frailer subset of older adults on the other hand, are unlikely to need or indeed be able to manage complex restorative dental procedures, and instead participants argued that the maintenance of a pain free functional dentition that will allow a sufficient nutritional intake is more appropriate. This approach involves optimal prevention, professional mechanical plaque removal and the occasional adjustment of broken restorations, all of which is within the scope of adequately trained dental care professionals, namely dental hygienists, and hygiene therapists. Whilst all participants were in favour of this approach, a few shared the concern that DCPs might be used inappropriately and asked to take too much responsibility or make decisions that they are not trained for, simply because other dental professionals do not want to manage this patient cohort. Some participants believed that this was because young entrants to the profession do not view this as an ‘attractive’ side of dentistry. This is an area that participants believe needs to be addressed, through better incentivisation and flexible commissioning of dental services to ensure that the workforce treats all members of the population equally and fairly.

A solution favoured by the participants and emphasised by some was that a team approach was essential where DCPs can work within their scope of practice with a dentist working closely alongside them. From this viewpoint, the UK dental skill mix is well suited to this approach that may not be reflected internationally. Participants further acknowledged the important role that Clinical Dental Technicians can play especially as many older patients have partial dentures, and still a significant number with complete dentures. Many participants reported the current NHS system as one of the major barriers to providing the appropriate dental care to older adults.

The last theme of education provided some reasons as to why younger dental professionals are seemingly not interested in providing dental care to older adults and is reflected in quantitative research. It was postulated that they do not feel adequately equipped to deal with the more complex care plans for older patients through insufficient training and education at the undergraduate level. As this is an increasingly important issue for the dental workforce, there has been much international research conducted aiming to investigate the current standard of training in Gerodontology globally. A recent UK study concluded that the training is not standardised and is insufficient for current and future needs. This is also importantly the case for the training delivered to UK dental hygiene therapists, which prior to this study has not been reported (Prosser G, Radford D, Louca C. Teaching of Gerodontology to Dental and Dental Hygiene Therapy students in the UK. Manuscript submitted for publication *Br Dent J*. 2022) [8]. Similarly in a recent international scoping review of the literature, large variations in the training were reported, and the review also highlighted the need for national and international guidelines to ensure mandatory inclusion of specific training in Gerodontology [17]. All participants reported the perennial issue of the overcrowded curriculum as the main barrier preventing a more thorough training and education in Gerodontology, however, all agreed that more emphasis needs to be put on both inter-professional and intra-professional education to achieve multidisciplinary care.

Further exploration into how appropriate education in Gerodontology can be incorporated efficiently and effectively into the curriculum of all dental professionals is urgently required, and this cannot be investigated through research of a quantitative nature alone. Instead, a conference of experts in this field would be most beneficial, to discuss the issues and make recommendations for the education of Gerodontology for future dental and dental care professional student cohorts in the UK.

A qualitative research methodology using semi-structured interviews was adopted as it is an appropriate approach when interviewing participants only once and provide reliable comparable qualitative data. The open-ended questions and flexibility of the interview structure also provided the opportunity for new opinions and ways of thinking to be uncovered [18]. This approach does, however, require a skilled interviewer and is unlikely to include a large enough sample to precisely estimate the views of the entire sample population [19].

Although the initial analysis of the interview transcripts was undertaken independently, there is a need to acknowledge researcher bias and that both researchers were from a dentist background with one relatively inexperienced and the second passionate about inter- and intra-disciplinary education and restorative care for the older adult. Also, all the participants interviewed were from a dental background, no patients or individuals involved with commissioning or planning of dental services were interviewed, and so the findings are less representative of the many involved stakeholders. Credibility of the research was achieved through member checking of the transcripts by respondents and dependability through description of the research steps from the start of the project to the development and reporting of the findings.

## Conclusion

Within the limitations of this qualitative study, the experts with an oversight of Gerodontology provided a detailed insight into the challenges in service delivery and educational opportunities for Gerodontology both in the UK and internationally. Whilst the challenges associated with the increasingly older population are complex and are of concern to dental professionals, this is compounded further by the vague nature of the term ‘Gerodontology’, which is poorly described and ambiguous owing to the diverse nature of this patient group. Although the participants stated there are various initiatives for ‘older people’ in the UK, several barriers are perceived to be still in place thus preventing older people from achieving optimal oral health and ultimately resulting in a reduced quality of life and general wellbeing. Participants in this study felt that independent older adults dental care needs are no different from working aged adults and should be treated in primary care, even if they have potentially more complex restorative dental needs and higher expectations than previous generations.

In contrast, the participants were significantly more concerned about the dental management of frailer older adults, typically those that are medically compromised, functionally dependent and often in residential settings. It is this older adult cohort the current system in the UK appears to be failing and that this needs to be addressed both by the profession and politicians. It is widely agreed that a multidisciplinary approach is essential, however, the dental profession does not promote itself well and should have a higher profile in the holistic care of older adults due to obvious links between general and oral health. The dental profession has the potential to hugely contribute to the quality of life for the older patient through the provision of regular prevention with the aim of maintaining a functional pain free dentition; however, again system failures and lack of incentivisation appear to be barriers that have been identified by this research.

## Supplementary information

Appendix 1 Topic Guide

## References

[1] Meurman JH, Mckenna G, Murtomaa H, Nakao M, Ogawa H, Walls A (2018). Managing our older population: the challenges ahead. J Dent Res.

[2] Thomson WM, Ma S (2014). An ageing population poses dental challenges. Singap Dent J.

[3] Petersen PE, Yamamoto T (2005). Improving the oral health of older people: the approach of the WHO Global Oral Health Programme. Community Dent Oral Epidemiol.

[4] Burke FM, Mckenna G (2010). Gerodontology: Now and the future. Dent Update.

[5] Issrani R, Ammanagi R, Keluskar V (2012). Geriatric dentistry - meet the need. Gerodontology.

[6] Kossioni A, Hajto-Bryk J, Maggi S, McKenna G, Petrovic M, Roller‐Wirnsberger RE (2018). An expert opinion from the European College of Gerodontology and the European Geriatric Medicine Society: European policy recommendations on oral Health in older adults. J Am Geriatr Soc.

[7] Petersen PE, Ogawa H (2018). Promoting oral health and quality of life of older people - the need for public health action. Oral Health Prev Dent.

[8] Prosser G, Radford D, Louca C. Teaching of gerodontology to dental and dental hygiene therapy students in the UK. Manuscript submitted for publication Br Dent J. 2022. 10.1038/s41415-022-4301-z 35689065

[9] General Dental Council (GDC). Guidance on Direct Access. 2019. https://www.gdc-uk.org/information-standards-guidance/standards-and-guidance/direct-access (accessed August 2021).

[10] Jaggee G, Dooey J, Gallagher J, Radford DR (2019). Bouncing on the fringes of the dental system: clinical dental technicians, a decade after their creation. Br. Dent. J..

[11] NHS England. Introductory Guide for Commissioning Dental Specialties. 2015. https://www.england.nhs.uk/commissioning/wp-content/uploads/sites/12/2015/09/intro-guide-comms-dent-specl.pdf (accessed September 2021).

[12] Office for National Statistics (ONS). Living longer: how our population is changing and why it matters. 2018. https://www.ons.gov.uk/peoplepopulationandcommunity/birthsdeathsandmarriages/ageing/articles/livinglongerhowourpopulationischangingandwhyitmatters/2018-08-13 (accessed November 2020).

[13] Age UK. Later life in the United Kingdom. 2019. https://www.ageuk.org.uk/globalassets/age-uk/documents/reports-and-publications/later_life_uk_factsheet.pdf?dtrk=true (accessed August 2021).

[14] Petersen PE, Kandelman D, Arpin S, Ogawa H (2010). Global oral health of older people - call for public health action. Community Dent Health.

[15] Kossioni A, Vanobbergen J, Newton J, Muller F, Heath R (2009). European College of Gerodontology: undergraduate curriculum guidelines in gerodontology. Gerodontology.

[16] NHS Health Education England. Advancing Dental Care: Education and Training Review Final Report. 2019. https://www.hee.nhs.uk/sites/default/files/documents/advancing_dental_care_final.pdf (accessed September 2020).

[17] Nilsson A, Young L, Glass B, Lee A (2021). Gerodontology in the dental school curriculum: A scoping review. Gerodontology..

[18] Qualitative Research Guidelines Project. Semi-Structured Interviews. 2006. http://www.qualres.org/HomeSemi-3629.html (accessed December 2020).

[19] Wholey JS, Hatry HP, Newcomer KE (2010). Handbook of practical program evaluation.

