# Supplementary material for: Potential educational and workforce strategies to meet the oral health challenges of an increasingly older population: a qualitative study
Source: BDJ Open. 2022 Mar 10;8:6. doi: 10.1038/s41405-022-00098-5 (PMC8907390; doi:10.1038/s41405-022-00098-5)
Supplement: Supplementary file 1 — Appendix 1 Topic Guide [file 41405_2022_98_MOESM1_ESM.docx]

**Appendix 1 Semi-structured Interview Topic Guide**

**Ageing population and dental challenges**

1. What are your opinions on the implications of the ageing population and associated dental and oral challenges?
2. During your career, have you personally encountered these challenges?
3. What impact do you think that these challenges might have on the future dental workforce?
4. Finally, do you think that the dental care of older people should be within the remit of special care dentistry?

**Role of Dental Therapists**

1. Do you think that dental care professionals, namely dental therapists, could play a significant role in addressing the unique dental needs of older adults?
2. What impact could direct access have on the provision of dental care to older people?
3. What are your opinions on a change to the NHS contract that would provide dental therapists with a performer number?
4. Do you think there might be a reaction from older people in response to seeing a dental therapist instead of dentist?
5. Do you think dental therapists being able to refer directly to clinical dental technicians would be of value?
6. Are dental therapists adequately trained to visit care homes to provide oral health care?

**Education in Gerodontology**

1. What are your opinions on the training in Gerodontology currently being delivered to undergraduate dental students in the UK?
2. What are your opinions on the training in Gerodontology currently being delivered to undergraduate dental therapy students?
3. What are your opinions on training and joint education, that encourage teamwork between DCP’s and dentists whilst students?
4. Do they think there is a difference between the teaching delivered to dental students and dental therapy students?
5. If you deem the teaching to be unsatisfactory, why do you think this is the case and what do you think can be done to improve it?
   1. What are your opinions on interprofessional training eg between dental care professionals and other health care professionals?

**Final remarks**

1. Finally, do you have any other comments or points that you would like to discuss further?
